# Assessment of digital payment for agents in mass chemoprevention campaigns: The Karangué Fay project in Senegal

**DOI:** 10.1371/journal.pdig.0000799

**Published:** 2025-10-16

**Authors:** Jean Augustin Diegane Tine, Amadou Yeri Camara, Aminata Diaw, Meissa Seck, Saliou Séne, Fatoumata Zahra Mohamed Mboup, Amadou Ibra Diallo, Fatoumata Bintou Diongue, Mouhamadou Faly Ba, Ibrahima Ndiaye, Souleymane Ndiaye, Adama Faye

**Affiliations:** 1 Institute of Health and Development (ISED), Cheikh Anta Diop University of Dakar (UCAD), Dakar, Senegal; 2 Ministry of Health and Social Action (MSAS), Dakar, Senegal; Jordan University of Science and Technology, JORDAN

## Abstract

The payment of healthcare agents is a critical component of organizing mass health campaigns. This study examined the effects of digital payments during seasonal malaria chemoprevention (SMC) campaigns in Senegal. A quasi-experimental three-arm before–after/here–elsewhere design was implemented between March and June 2023: mandatory digital payment in Kounkané, voluntary in Koussanar, and cash-based control in Bantaco. Mixed methods were employed, and ethical approval was granted by Senegal’s National Ethics Committee (CNERS). A total of 299 agents participated, with 181 surveyed before and 118 after the intervention. Participants were distributed across Kounkané (48.8%), Koussanar (35.8%), and Bantaco (15.4%). Community health workers comprised the majority (90.9%). Median age was 32 years, with a median tenure of three years; 50.8% were male and 65.2% married. All agents owned at least one mobile money account, with Wave (96%) and Orange Money (90%) being the most common. Reliability criteria included security (95%), ease of use (90.3%), and cash availability (79.2%). Karangué Fay digital payments were perceived as secure (82.4%), reliable (83.1%), faster (78.2%), transparent (91.3%), and confidential (95.2%). Compared to cash, digital payments were significantly preferred for transaction security, speed, and usability (p < 0.001). Qualitative interviews highlighted traceability, transparency, and efficiency but noted limitations linked to internet connectivity. Digital payments were highly acceptable to SMC agents, improving engagement and performance. They also contributed to better campaign coverage across different implementation phases, underscoring their potential to strengthen health systems in low-resource settings.

## Author summary

Timely and transparent payment of healthcare agents is critical for mass health campaigns. This study assessed the effects of digital payments during seasonal malaria chemoprevention (SMC) campaigns in Senegal. A quasi-experimental three-arm before–after/here–elsewhere design was implemented from March to June 2023, covering a mandatory digital payment zone (Kounkané), a voluntary zone (Koussanar), and a control zone without digital payment (Bantaco). Mixed methods were employed, and ethical approval was obtained from Senegal’s National Ethics Committee (CNERS). A total of 299 agents participated, with 181 surveyed before and 118 after the intervention. Most were community health workers (90.9%), median age 32 years, with a median tenure of three years; all owned at least one mobile money account. Orange Money (90%) and Wave (96%) were most frequently used. Key criteria for selecting an operator included security (95%), ease of use (90.3%), and cash availability (79.2%). Agents widely perceived digital payments as secure (82.5%), reliable (83.1%), transparent (91.3%), and confidential (95.2%), offering faster transactions compared to cash (p < 0.001). Qualitative findings underscored benefits of traceability and efficiency, though dependent on internet stability. Digital payments demonstrated high acceptability, strengthening agent engagement, improving transparency, and supporting effective SMC campaign delivery.

## 1. Introduction

The financial autonomy of healthcare providers affects the quality of service delivery and the healthcare system’s ability to meet users’ needs. When healthcare providers have greater decision-making authority across different domains, they gain more flexibility to adapt to purchasing and payment incentives, thereby enhancing the effectiveness of these incentives [1]. However, provider autonomy must be accompanied by management capacity, access to information, and accountability [2].

In Senegal, national and regional campaigns led by the Ministry of Health and Social Action (MSAS) engage a large number of volunteer community members who receive financial compensation through allowances. These payments are often made in cash, requiring the transport of substantial sums across regions and exposing the process to serious logistical and security risks [3]. Since the early 2010s, many international actors have highlighted the potential of mobile phones in African healthcare and have launched various mobile-health (mHealth) initiatives across the continent [4,5].

This study aims to address the limitations of traditional cash payment systems in mass campaigns by introducing a digital payment system in a rural sub-Saharan setting. The Seasonal Malaria Chemoprevention (SMC) campaign was used as a pilot intervention in three regions of southern Senegal (Kolda, Tambacounda, and Kédougou). SMC is an effective public health intervention for reducing the burden of malaria in high-risk regions, particularly for children who are more vulnerable to severe *Plasmodium falciparum* infections [6]. However, it is crucial that SMC is implemented in accordance with World Health Organization (WHO) guidelines and national protocols to prevent drug resistance and adverse effects. To be effective in reducing malaria transmission, it should be integrated with other malaria control interventions, such as the use of insecticide-treated mosquito nets, early diagnosis and treatment of malaria, indoor residual insecticide spraying, and environmental sanitation initiatives [7].

The overall objective of this research is to assess the effects of digital payments in seasonal malaria chemoprevention campaigns in Senegal. Specifically, the study aims to: i) Map payment systems and financial transaction circuits in the study areas; ii) Analyze the perceptions and behaviors of healthcare actors regarding digital payments in health interventions such as SMC; iii) Evaluate the experimental digital payment system “Karangué Fay” used during the SMC campaign in Senegal; iv) Assess the effects of the digital payment strategy on the implementation of the 2023 SMC campaign in Senegal.

## 2. Methods

### 2.1. Study area

Malaria is an endemic disease in Senegal, exposing the entire population to its risks. In 2020, the country accounted for 0.7% of global malaria-related deaths [8]. Between 2017 and 2020, recorded malaria cases decreased by 4.4%, from 52 to 50 cases per 1,000 people at risk. However, during the same period, malaria-related mortality slightly increased by 1.8%, rising from 0.24 to 0.245 deaths per 1,000 people at risk [9].

To combat this public health issue, Senegal’s National Malaria Control Program (PNLP) has implemented various interventions, including the distribution of long-lasting insecticide-treated nets (LLINs), community-based malaria management (PECADOM), and school-based initiatives such as PECA-École and PECA-Daara in traditional and Quranic schools. Another key intervention is Seasonal Malaria Chemoprevention (SMC). Recommended by the World Health Organization (WHO) in 2012, SMC targets children up to the age of ten. Senegal has been a major research site for SMC, initially targeting children under five and later expanding to those under ten.

SMC campaigns are implemented in sixteen districts across the five most affected regions (*Kédougou, Kolda, Tambacounda, Kaolack, and Diourbel*). These interventions cover all districts in Kolda, Kédougou, and Tambacounda, as well as the districts of Touba, Diourbel, and Kaolack. In 2021, monthly treatment rounds were conducted over four months in Kédougou and three months in other target regions. Due to the length of the transmission season in these areas, an additional monthly round was introduced in 2022 to improve coverage during the transmission period [10].

In Senegal, the mobile telecommunications sector generated 428 billion CFA francs (1 USD = 600 CFA) in 2020, down from 467 billion CFA francs in 2019, marking a decline of 8.39%. This market was also affected by the COVID-19 pandemic. Despite this, the number of mobile phone lines continued to grow in 2020, reaching 19,078,948 lines, a 6.7% increase compared to 2019 [11].

According to the University of Sherbrooke, internet usage in Senegal grew from 1% in 2002 to 42.6% in 2020 [12]. Additionally, World Bank data indicates that Senegal has one of the highest smartphone adoption rates in West Africa (35.6%), compared to a regional average of 28%. Mobile payment adoption in businesses varies by company size, with 2% in small enterprises, 2.5% in medium enterprises, and 2.8% in large enterprises [13].

The USAID Global Health Finance and Governance (HFG) Project promotes mobile money usage to strengthen health systems [14]. As part of this study, three districts in southern Senegal were selected [15]: Kounkané (Kolda), Koussanar (Tambacounda), Bantaco (Kédougou).

### 2.2. Study design and recruitment of participants

This is a quasi-experimental study using a before-after/here-elsewhere three-arm design: (i) the first intervention arm implemented mandatory digital payments in Kounkané, (ii) the second intervention arm allowed voluntary digital payments in Koussanar and (iii) the control arm used no digital payment in Bantaco.

Participant recruitment was exhaustive at the level of each health post. All agents involved in the Seasonal Malaria Chemoprevention (SMC) program at the selected health posts were included in the study. Due to the limited number of eligible personnel actively participating in the SMC delivery at the health post level, it was feasible to include the entire population of interest without requiring individual-level sampling.

However, site selection was guided by a combination of random and purposive sampling approaches. The health posts of Kounkané and Koussanar were chosen through random sampling, with one post drawn from each of the two regions where the SMC program was implemented. The Bantaco health post was selected based on a purposive recommendation by the national SMC program coordinator, who identified the site as operationally appropriate for the control arm.

### 2.3. Data collection and analysis

A mixed-method approach combining both qualitative and quantitative methodologies was used for data collection.

The data collectors were selected based on their academic background (studies in epidemiology, sociology, or health sciences) and their prior experience in field surveys. They completed a three-day training focused on the Karangué Fay project and the use of data collection tools.

Quantitative data were collected through structured individual interviews with all agents participating in the Seasonal Malaria Chemoprevention (SMC) campaign across the study sites. Data collection was conducted both before and after the deployment of the Karangué Fay digital payment platform. Data collectors entered participants’ responses directly into Android tablets using the ODK Collect application, ensuring real-time, accurate, and secure data capture.

Categorical variables were described using absolute and relative frequencies. Continuous variables were described using mean and standard deviation for parametric distributions and median and interquartile range (IQR) for non-parametric distributions. All statistical analyses were performed at a significance level of α = 0.05. Based on the type and distribution of the variables, we used: (i) the Kruskal-Wallis rank sum test to compare medians across groups; (ii) Pearson’s Chi-squared test for categorical variables with sufficient expected frequencies; and (iii) Fisher’s exact test for categorical data with small or sparse samples. The RStudio 4.3.3 software [16] was used for data analysis.

Qualitative data collection employed three methods: Semi-structured individual interviews, Focus group discussions and Informal interviews.

The qualitative data collection followed an iterative process, including an initial exploratory visit and a post-intervention visit. In parallel, several informal interviews were conducted using an empirical-inductive approach. Qualitative data collected in the field were processed using Nvivo 12 software [17].

### 2.4. Ethical considerations

Ethical approval was obtained from the Senegal National Ethics Committee for Health Research (CNERS) [18] before field activities began (approval number 0209 MSAS/CNERS/SP). Additionally, authorization was obtained from the Ministry of Health and Social Action through the National Malaria Control Program (PNLP) before the study commenced. The medical authorities of the Kolda, Tambacounda, and Kédougou regions, as well as district health officials, were briefed on the project and actively involved in its implementation. Participation in this study was voluntary. Informed consent was obtained from all participants. An information sheet was provided to each participant before signing the consent form. The data were stored in secure databases protected by access codes. All data were anonymized and treated with strict confidentiality throughout the study.

### 2.5. Description of the Karangué Fay project

The Karangué Fay project was submitted under the Digital Health Payment Initiative and Research (DHPI-R) project [19], funded by “Bill and Melinda Gates Foundation” and aims to generate evidence on digital payment for rural health workers and establish its impact on the delivery of health campaigns in sub-Saharan Africa.

As part of this project, the Karangué Fay (KF) platform was developed through a participatory and iterative approach involving stakeholders from the Ministry of Health and Social Action (MSAS), the University Cheikh Anta Diop (UCAD), and community actors between March and June 2023.

The platform is a web application, developed with local expertise, capable of enabling money transfers through three payment methods: Wave [20], Orange Money [21], and Free Money [22]. It also allows transaction tracking and payment status generation while ensuring multi-level security, which will be described later. Access to the platform was provided through the URL www.karanguefay.sn, after pre-registration of users by the system developer. Authorization levels varied among users based on their roles and responsibilities within the healthcare system. Regional Health Directors (DRS *(directeur régional de la santé)*) and Regional Managers (GR *(gestionnaire de la région médicale)*) were responsible for overseeing district-level operations, while District Chief Medical Officers (MCD *(médecin chef de district)*) supervised health post payments remotely from their offices. District Managers (GD *(gestionnaire du district sanitaire)*) managed the platform by updating the list of healthcare agents eligible for payment. At the health post level, Head Nurses (ICP *(infirmier chef de poste)*) ensured the accuracy of payment requests by verifying names and amounts before approval. The Karangué Fay platform administrator had full access rights, allowing them to monitor transactions and cancel duplicate payments when necessary. Access to the platform was secured through a two-step authentication process: (i) Email login and password entry, (ii) A confirmation code sent via SMS.

The beneficiary’s information including first name, last name, national identification number, phone number, and preferred mobile money operator, was recorded on the platform.

Afterwards, the transfer amount was assigned to each beneficiary. A payment confirmation message was sent via text message, stating: « ***Vous venez de recevoir XX 000 FCFA via Orange Money (ou Wave ou Free Money), envoyé par Karangué Fay*** » (“You have received XX,000 FCFA via Orange Money (or Wave or Free Money), sent by Karangué Fay”).

A payment report was generated in PDF format, including the logos of partner organizations and validated activity titles. This report was then sent via email to the various managers and was also available for direct download from the platform.

## 3. Results

The total number of agents who participated in the study was 299, with 181 in the pre-intervention phase and 118 in the post-intervention phase. This difference is explained by the exclusion of certain SMC agents following the results obtained during the preliminary training before their field enrollment. The participants were residents of Kounkané at 48.8%, then of Koussanar at 35.8% and of Bantako 15.4%. The key actors involved were predominantly community health workers (90.9%), followed by supervisors (5.4%). Regarding marital status, 65.2% of the participants were married. Men constituted a slight majority, representing 50.8% of the participants. The median age of participants was 32 years, and their median tenure in their roles was 3 years.

The Table 1 presents a comparison of the sociodemographic characteristics of agents across the three study zones (Bantaco, Kounkané, Koussanar) prior to the digital intervention. No statistically significant differences were observed with respect to sex (p = 0.94), marital status (p = 0.80), education level (p = 0.39), ownership of a telephone (p = 0.95), mobile phone (p = 0.91), bank account (p = 0.58), or knowledge of digital payment methods (p = 0.44). The median age was also comparable across the three zones (p = 0.23), indicating a similar distribution of age groups. These findings suggest a general baseline homogeneity between the groups at the start of the study, thereby providing a solid foundation for subsequent comparison and evaluation of the impact of the Karangué Fay digital payment intervention.

**Table 1:** Comparison of sociodemographic variables between study zones (Bantaco, Kounkané, Koussanar) before implementation of the Karangué Fay platform.

Characteristic	Bantako (n = 29)	Kounkane (n = 78)	Koussanar (n = 74)	p-value
**Sex**				0.299
Female	11 (37.9%)	36 (46.2%)	40 (54.1%)	
Male	18 (62.1%)	42 (53.8%)	34 (45.9%)	
**Marital status**				0.299
No	8 (27.6%)	27 (34.6%)	32 (43.2%)	
Yes	21 (72.4%)	51 (65.4%)	42 (56.8%)	
**Education**				0.900
No	1 (3.4%)	2 (2.6%)	3 (4.1%)	
Yes	28 (96.6%)	76 (97.4%)	71 (95.9%)	
**Telephone**				0.599
No	0 (0.0%)	0 (0.0%)	1 (1.4%)	
Yes	29 (100.0%)	78 (100.0%)	73 (98.6%)	
**Mobile phone**				0.599
No	0 (0.0%)	0 (0.0%)	1 (1.4%)	
Yes	29 (100.0%)	78 (100.0%)	73 (98.6%)	
**Email**				0.599
No	15 (51.7%)	37 (47.4%)	41 (55.4%)	
Yes	14 (48.3%)	41 (52.6%)	33 (44.6%)	
**Bank account**				0.299
No	20 (69.0%)	44 (56.4%)	50 (67.6%)	
Yes	9 (31.0%)	34 (43.6%)	24 (32.4%)	
**Knowledge of digital payment methods**				0.599
No	0 (0.0%)	0 (0.0%)	1 (1.4%)	
Yes	29 (100.0%)	78 (100.0%)	73 (98.6%)	
**Age (Median (Q1, Q3))**	33 (26, 42)	31 (27, 35)	32 (22, 41)	0.699

### 3.1. Mapping of payment systems and financial transaction circuits

The mapping of digital platforms allowed us to understand the feasibility of digital payments in the study area. Mobile phones were the most commonly used tool among SMC agents, with 99% of healthcare agents owning a functional mobile phone. For the Karangué Fay project, having a mobile phone was essential for payment verification. A total of 98% of agents were already subscribed to a mobile network operator that enabled digital money transactions. Wave was the preferred payment method across all intervention areas, and cash withdrawal was the primary transaction conducted. Bank account coverage was low across the three research sites. The Table 2 presents the availability of digital payment options.

**Table 2:** Comparison of participants’ access to and use of mobile and financial services before and after the intervention.

Characteristics	Pre-intervention (n = 181)	Post-intervention (n = 118)	Total (N = 299)	P-value
**Functional Mobile Phone**				**0.9**
No	1 (0.6%)	1 (0.8%)	2 (0.7%)	
Yes	180 (99.4%)	117 (99.2%)	297 (99.3%)	
**Email**				**0.01**
No	93 (51.4%)	78 (66.1%)	171 (57.2%)	
Yes	88 (48.6%)	40 (33.9%)	128 (42.8%)	
**Subscription to an Operator**				**0.9**
No	3 (1.7%)	2 (1.7%)	5 (1.7%)	
Yes	178 (98.3%)	116 (98.3%)	294 (98.3%)	
**Subscription to Orange Money**				**0.001**
No	8 (4.5%)	21 (17.8%)	29 (9.8%)	
Yes	170 (95.5%)	97 (82.2%)	267 (90.2%)	
**Subscription to Wave**				**0.6**
No	6 (3.4%)	6 (5.1%)	16 (5.4%)	
Yes	172 (96.6%)	112 (94.9%)	284 (94.6%)	
**Subscription to Free Money**				**0.2**
No	146 (82.0%)	103 (87.3%)	249 (84.1%)	
Yes	32 (18.0%)	15 (12.7%)	47 (15.9%)	
**Preferred Network**				**0.9**
Orange Money	33 (18.6%)	22 (19.0%)	55 (18.8%)	
Wave	143 (80.8%)	94 (81.0%)	237 (80.9%)	
Free Money	1 (0.6%)	0 (0.0%)	1 (0.3%)	
**Types of Regular Transactions**				**0.9**
Sending	47 (26.0%)	27 (22.9%)	74 (24.7%)	
Withdrawal	131 (72.4%)	89 (75.4%)	220 (73.6%)	
None	3 (1.6%)	2 (1.7%)	5 (1.7%)	
**Bank Account**				**0.05**
No	114 (62.9%)	87 (73.7%)	201 (67.2%)	
Yes	67 (37.1%)	31 (26.3%)	98 (32.8%)	

### 3.2. Perceptions of the functioning of different payment methods

**Fig 1:**
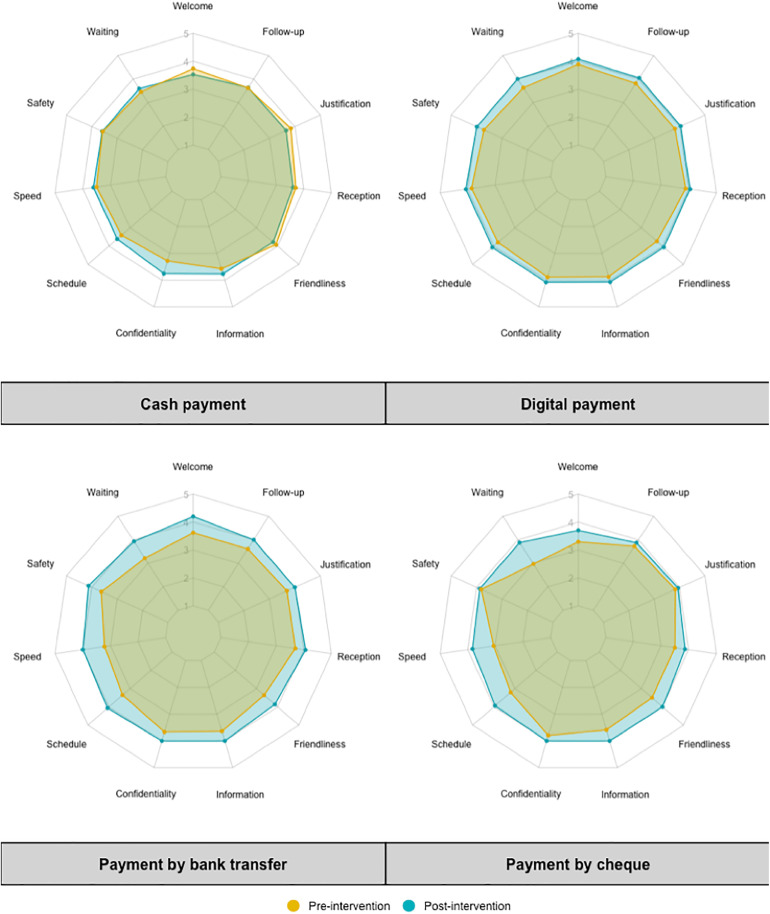
Synoptic representation of the appreciation levels of the qualities of different payment methods throughout the project.

In the study area, several payment methods were identified: cash payment, bank transfer, check payment, and digital payment. Among these options, cash payment remains the most traditional method. Fig 1 illustrates the evolution of beneficiaries’ perceptions regarding these different payment methods before and after the intervention. The results highlight that cash payments are perceived as less confidential, with longer waiting times. Payments made via bank transfer or check are also considered less efficient due to the time constraints imposed by banks’ opening and closing hours.

Conversely, digital payment received the highest satisfaction scores across the eleven evaluated dimensions, both before and after the intervention. Respondents particularly emphasized speed, convenience (notably the user-friendliness of the process), and traceability (ease of justification) as the main reasons for their satisfaction with this payment method.

Qualitative interviews provided deeper insights into perceptions of different payment methods. Although cash payment is widely used and well understood by managers, users acknowledge that it has significant drawbacks, such as increased vulnerability to theft and money loss. However, this payment method avoids the risks associated with online fraud, which is a notable advantage. Bank transfers and check payments are generally perceived as more secure options. Nevertheless, these payment methods involve operational constraints, including extended waiting times at banks. Additionally, difficulties may arise due to administrative requirements, such as signatures and identification documents. A nurse from Kounkané shared: *«* ***J’ai souvent rencontré des erreurs dans l’écriture de mon prénom, ce qui m’a causé des difficultés pour percevoir mon argent à la banque*** *» (“I have often encountered errors in the spelling of my first name, which caused difficulties in receiving my money at the bank.”).* Other participants also reported input errors that led to similar complications in the banking process. Finally, digital payments, while equipped with advanced security protocols, require a reliable transfer network to ensure their efficiency and accessibility.

### 3.3. Assessment of the costs of different payment methods

**Fig 2:**
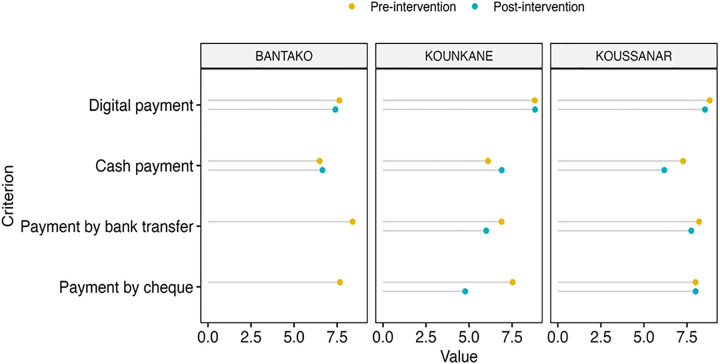
Preferential assessment of payment methods based on costs (direct and indirect) on a scale from 0 to 10.

This study examined perceptions of the costs associated with different payment methods used in health campaigns in Senegal. Cash payments were found to incur additional indirect costs, including transportation expenses and income loss due to waiting times. Bank transactions involved account maintenance fees and the purchase of transaction tools, while digital payments required access to a mobile phone and an active subscription, with some platforms applying transaction or conversion fees. Using a satisfaction scale from 0 to 10, participants assessed the perceived cost burden across sites (Bantaco, Kounkané, and Koussanar). Results (Fig 2) showed that digital payments were perceived as more cost-effective following the intervention, especially in Kounkané and Koussanar, suggesting improved acceptability. In contrast, cash and check payments remained associated with high perceived costs, particularly due to the need to travel to collect funds. Bank transfers were also rated less favorably in Koussanar, where perceived costs increased. The findings highlight the economic advantage of digital payment adoption.

### 3.4. Assessment of user satisfaction with the Karangué Fay platform

The project assessed preferences for digital payment across multiple dimensions. Head Nurses (ICP) and community health workers particularly appreciated the time and resource savings offered by this payment method. Since transactions were conducted remotely, the ICPs were able to continue their healthcare activities without interruption, while community health workers did not have to pause their usual tasks to receive their payments. Moreover, beneficiaries did not face difficulties related to finding change, a constraint that often kept them waiting late at health posts. Most respondents reported having waited several days to receive their payments; some even had to travel multiple times before receiving their funds. One community health worker shared: ***« Oui, il faut aller au poste de santé de Koussanar pour recevoir le paiement. Je n’avais pas de problème parce que j’habite à 4 kilomètres en plus j’ai une moto donc la distance ne me pose pas problème. Mais avec le paiement en espèce on a souvent des difficultés parce qu’il nous arrive de faire le déplacement jusqu’au poste et l’argent n’est pas disponible »*** (*“Yes, I had to go to the Koussanar health post to receive the payment. I didn’t have a problem because I live 4 kilometers away, and I have a motorcycle, so the distance wasn’t an issue for me. But with cash payments, we often face difficulties because sometimes we travel to the post, and the money isn’t available”).*

These observations confirm that digital payment facilitates better time and financial resource management, meeting beneficiaries’ expectations while improving their efficiency in carrying out their tasks. A community supervisor shared in an individual interview: ***«Oui on avait un gain de temps, personnellement, le paiement numérique m’a beaucoup arrangé parce que avec le paiement en espèce, parfois, c’est moi qui récupérait les fonds au niveau du district, parfois aussi c’est moi qui faisait le paiement de ce fait après la campagne je restais plus de trois jours à gérer les paiements. Mais lors de ce dernier passage, j’avais terminé le travail samedi et le dimanche, j’ai déposé les matériels dans le poste c’est à ce moment qu’on m’a envoyé le message du paiement »*** *(“Yes, we saved time. Personally, digital payment was very convenient for me because with cash payments, sometimes I was the one collecting the funds from the district, and at times, I was also responsible for making the payments. As a result, after the campaign, I would spend over three days managing payments. But during this last round, I finished work on Saturday, and on Sunday, I dropped off the materials at the post, at that moment, I received the payment notification”).*

The overall satisfaction assessment regarding the digital payment system implemented by Karangué Fay revealed that 88.9% of users reported being completely satisfied. This satisfaction rate was 87% in Kounkané and 90.5% in Koussanar (see Table 3). Additionally, the overall acceptance of using the platform for future payments was 97.3%, with 97.4% in Kounkané and 97.2% in Koussanar.

**Table 3:** User satisfaction and acceptability of the Karangué Fay digital payment platform in the Kounkané and Koussanar health districts.

Items	Kounkané (n = 78)	Koussanar (n = 74)	Total (N = 152)
**Satisfaction by Dimension**			
Reliable	65 (83.0%)	62 (83.2%)	126 (83.1%)
Secure	63 (81.1%)	62 (83.8%)	125 (82.4%)
Fast	60 (77.3%)	59 (79.1%)	119 (78.2%)
Transparent	70 (90.3%)	68 (92.2%)	139 (91.2%)
Confidential	73 (94.0%)	71 (96.4%)	145 (95.2%)
**Overall Satisfaction**			
Very Satisfied	68 (87.2%)	67 (90.5%)	135 (88.9%)
Satisfied	4 (5.1%)	5 (6.8%)	9 (5.9%)
Somewhat Satisfied	4 (5.1%)	2 (2.7%)	6 (3.9%)
Not Satisfied	2 (2.6%)	0 (0.0%)	2 (1.3%)
Not at All Satisfied	0 (0.0%)	0 (0.0%)	0 (0.0%)
**Acceptability of Using the KF Platform for Future Campaigns**			
Yes	76 (97.4%)	72 (97.2%)	148 (97.3%)
No Opinion	2 (2.6%)	1 (1.4%)	3 (2.0%)
No	0 (0.0%)	1 (1.4%)	1 (0.7%)

The findings did not identify any specific individual characteristics that were significantly associated with the adoption of digital payments in SMC campaigns. Rather, adherence to this payment modality appears to be driven primarily by technical and organizational factors rather than by individual-level attributes.

Despite the predominantly positive feedback collected from various stakeholders, certain limitations were identified at the two sites where payments were processed. The main challenges involved transaction failures and cancellations. In Koussanar, respondents reported six cases of transaction failures. According to the information gathered, these failures were attributed to: two number entry errors, one inactive phone number, and three cases where community health workers had selected a payment operator without having an active account with that operator.

The ICP of Koussanar explained: **« Ce sont des petits détails à mon avis, car ils étaient liés à des problèmes de remplissage. Nous avons constaté des erreurs dans la saisie des numéros, souvent dues à une méconnaissance des relais, par exemple lorsqu’ils indiquaient des numéros incorrects ou commençaient par 77 au lieu de 76. »** (*“In my opinion, these are minor details, as they were related to form-filling issues. We observed errors in number entry, often due to a lack of familiarity among community health workers, for instance, when they provided incorrect numbers or mistakenly used 77 instead of 76 at the beginning of a number.”).*

These findings highlight the critical importance of raising awareness among stakeholders about the digital payment process. Such awareness efforts are essential to ensuring the successful implementation of this payment method during large-scale health campaigns.

### 3.5. Impact of the digital payment strategy on the implementation of the 2023 SMC campaign in Senegal

Formally attributing a reduction in malaria-related morbidity to the Karangué Fay digital payment initiative remains complex due to the multifactorial nature of disease outcomes. However, the project’s potential contribution can be inferred through operational indicators, particularly the coverage rates of Seasonal Malaria Chemoprevention (SMC) activities in the intervention sites. Table 4 presents data collected from the health posts in Kounkané and Koussanar, where the project was implemented, compared to the control site in Bantako. The results indicate consistently higher SMC coverage rates in both intervention areas relative to the control. Notably, Kounkané achieved 99% coverage across all three rounds of directly observed treatment (DOT), with 100% of targeted children receiving all three doses. Koussanar showed similarly strong performance, while Bantako, despite performing well, recorded slightly lower coverage and completion rates. These findings suggest that the implementation of digital payment systems, by improving timeliness, traceability, and administrative efficiency, may have positively influenced community health worker motivation and engagement, thereby enhancing the overall delivery and completeness of the public health campaign.

**Table 4:** Performance of the 2023 SMC campaign: Coverage rates and completion of treatment across study sites (Kounkané, Koussanar, Bantako).

SMC Performance Indicators	Kounkané	Koussanar	Bantako
Registered Children/ Treated Children	386/390	4202/4353	1414/1485
Coverage for DOT-1	99%	96.5%	95.2%
Coverage for DOT-2	99%	96.5%	95.4%
Coverage for DOT-3	99%	96.5%	95.4%
Coverage for All 3 Rounds	100%	100%	99.5%

DOT-1: Directly Observed Treatment – First Round.

## 4. Discussion

Over the past decade, digital technology has gained renewed interest with the widespread adoption of mobile phones and mobile internet across the African continent. Africa now hosts the largest number of mobile development programs, or *mDevelopment*, positioning itself as a *new digital frontier* driven by its mobile revolution [23,24].

The digitalization of payments involves the complete dematerialization of financial transactions, including both revenue collection and expenditures [25]. A digital environment necessitates a strong commitment to providing clients with transparent information about financial products, particularly when such information is accessible only through electronic means, such as mobile technology [26]. This was a key consideration in the Karangué Fay pilot project, whose preliminary objective was to assess the digital environment of the study area and evaluate the feasibility of implementing digital payment systems.

The study zones are rural areas with generally low living standards. However, mobile phone ownership was found to be 99%, with 98% of participants subscribed to a mobile money operator. Moreover, 74% of participants had prior experience with digital payments, particularly for withdrawals. The rapid expansion of mobile telephony is closely linked to the limited penetration of fixed-line telephony in sub-Saharan African countries (3% in Senegal, 1.2% in Côte d’Ivoire, and 0.9% in Burkina Faso) [5]. In 2020, 46.2% of the Senegalese population had internet access, though usage remained concentrated in major urban areas [11,12]. Most users accessed the internet via 3G mobile networks, but limitations in 3G and 4G coverage, as well as the high cost of internet access and mobile communication services, restricted usage to wealthier social classes [5,12].

Digital health technologies have the potential to be cost-effective tools that improve healthcare quality, optimize expenditures, and enhance patient-provider interactions [3,27,28]. In many African countries where bank account penetration is relatively low (33% in our study), mobile banking presents a significant opportunity for digital health [29]. It enables individuals to pay for healthcare expenses via mobile money while also facilitating the remuneration of healthcare workers.

In this project, overall satisfaction with digital payment was 94.8%, with 88.9% of users reporting complete satisfaction. Digital transactions were perceived as less costly than the indirect expenses associated with other payment methods, such as cash or bank transfers. Acceptance of the Karangué Fay digital payment platform was 97.3%, and health workers expressed a strong preference for continuing this payment method in future malaria chemoprevention campaigns.

Since 2021, Senegal has launched the “eSanté” initiative, a national program for the digitalization of the healthcare system, covering both healthcare professionals and patients over the 2022–2027 period [30]. This program aims to integrate digital technologies and platforms into the healthcare system to enhance service delivery and health governance.

This study found no significant socio-cultural or economic barriers to the adoption of digital payment by healthcare workers. However, structural challenges were identified. Six transaction failures out of 154 transfers were documented, primarily due to errors in beneficiary information (e.g., incorrect phone numbers or selection of an inactive mobile money operator). Additional challenges included internet connectivity issues and the availability of cash at mobile money withdrawal points.

For digital payment systems to be fully effective, seamless financial transaction management is essential. This requires a well-coordinated synergy between internet service providers, mobile money operators, local merchants, and end-users [31].

The adoption of mobile financial services in insurance and healthcare is closely linked to building a sustainable and profitable mobile economy in Africa, where 90% of users purchase mobile credit on a pay-as-you-go basis and frequently switch service providers. In this context, eHealth solutions can serve as loyalty programs, helping service providers retain customers in an increasingly competitive and unstable market [32].

The high acceptability of digital payments among health workers involved in SMC campaigns contributed to improved campaign coverage across multiple implementation rounds. This, in turn, strengthened the engagement of community health workers and enhanced their performance.

The World Health Organization’s (WHO) Global Strategy on Digital Health (2020–2025) states that:

*“Digital health must be an integral part of healthcare priorities, ensuring ethical, safe, reliable, equitable, and sustainable benefits for individuals. It should be developed based on principles of transparency, accessibility, scalability, replicability, interoperability, privacy, security, and confidentiality”* [26]. These are precisely the key attributes and values expected of digital payment systems.

Equity considerations are central to the sustainable adoption of digital health payments [3]. Our findings highlight the need to address potential disparities linked to gender, literacy, and rural connectivity. Women and individuals with limited literacy skills may face additional barriers in accessing and using digital platforms [28], while rural areas often experience network instability and limited infrastructure. To mitigate these risks, safeguards such as simplified user interfaces, targeted training sessions, and complementary support mechanisms are essential. Ensuring that digital health interventions are inclusive and accessible to the most vulnerable groups will be critical to preventing exclusion and promoting equitable health outcomes [29].

In light of the findings, the broader adoption of digital payments in health systems, particularly in the context of mass health campaigns, requires a series of enabling conditions to ensure long-term success. These include strong political commitment to institutionalize digital payment mechanisms within national health strategies, the reinforcement of digital literacy and technical skills among health personnel, and targeted investments to improve network infrastructure, especially in underserved and rural areas. Furthermore, the integration of digital tools into the Ministry of Health’s operational frameworks, including budgeting and planning processes, is essential to support sustained implementation. These policy and operational enablers are critical to achieving a scalable and resilient digital health payment ecosystem in Senegal and similar contexts.

### 4.1. Study limitations

This study has several limitations, including the risk of selection bias due to purposive sampling of the control site, the absence of a formal cost-effectiveness analysis, and the lack of objective measures of the digital divide. The short observation period also limited the assessment of long-term impacts, while the reliance on user perceptions may have introduced reporting bias. Moreover, analyses were univariate and exploratory, with no adjustments for confounders or multiple testing. Operational challenges such as network reliability, electricity supply, training requirements, and the sustainability of per diem-based payments further constrain scalability.

## 5. Conclusion

In its WHA58.28 resolution on eHealth, the World Health Organization (WHO) urged Member States *“to consider developing a long-term strategic plan for designing and implementing eHealth services across various areas of the health sector [...] to promote equitable, affordable, and universal access to their benefits”* [33].

According to the Ministry of Health and Social Action (MSAS), Senegal’s eHealth digitalization program aims to provide users with accurate health information, enhance the quality and safety of care, expand the range of medical specialties available to healthcare workers, and strengthen the connection between patients and healthcare providers [34].

The transition to digital health in Senegal represents a promising opportunity, yet it requires a comprehensive understanding of the challenges associated with this shift. Our study, which examines the use of digital payments in community health activities, highlights several key insights. It reveals that health providers overwhelmingly own functional mobile phones and are predominantly subscribed to mobile money services, particularly Wave and Orange Money.

Digital payments are perceived as fast, secure, traceable, and convenient, although their efficiency remains contingent upon network coverage and the availability of withdrawal points. The Karangué Fay platform, considered reliable, transparent, and confidential, has enhanced the acceptability of digital payments and contributed to improving the performance of community health activities, particularly in the project implementation areas.

These findings underscore the potential of digital tools to enhance the effectiveness of health interventions in Senegal.
